# Predictors of Academic and Non-Academic Termination From Medical Schools

**DOI:** 10.7759/cureus.98889

**Published:** 2025-12-10

**Authors:** Kenneth D Royal, Andrea Carpentieri, Robert Santos, David Stenulson

**Affiliations:** 1 Admissions Analytics and Insights, Association of American Medical Colleges, Washington, DC, USA

**Keywords:** academic dismissal, academic metrics, academic withdrawal, grade point average (gpa), mcat, medical school admissions, medical student outcomes, non-academic dismissal, preview, professionalism

## Abstract

Background and objective

Termination from medical school is relatively uncommon but typically results from either withdrawal or dismissal. Although the number of individuals terminated for academic and non-academic reasons annually is quite small, the collective impact is substantial and has significant implications for students, medical schools, public health, and the physician workforce. While some types of termination may be impossible to predict, those linked to academic performance or non-academic factors such as professionalism may be more predictable. This study aimed to examine how admissions metrics compare between medical students terminated for academic or non-academic reasons versus a baseline group consisting of all other matriculating students and recent graduates.

Methods

This study examined aggregate national data for student cohorts matriculating into traditional MD-granting degree programs during the 2021-2024 academic years. We examined only those students who 1) withdrew for academic reasons; 2) were dismissed for academic reasons; or 3) were dismissed for non-academic reasons. Scores from three common admissions metrics (Medical College Admission Test (MCAT®) scores, undergraduate grade point average (uGPA), the Association of American Medical Colleges (AAMC) PREview® Professional Readiness Exam) were compared between terminated students and the baseline group during this period.

Results

The 169 students who withdrew for academic reasons had significantly lower MCAT scores and uGPAs than the baseline group. The differences in MCAT scores resulted in a large effect size, whereas differences in uGPA led to a medium effect size. The differences in PREview scores, although not statistically significantly different, did present a medium effect size. The 193 students dismissed for academic reasons also had significantly lower median MCAT scores and uGPAs than the baseline group, and the contrast in scores had large effect sizes for both measures. The PREview scores were identical between the academically dismissed and baseline groups, indicating no meaningful difference. The 42 students dismissed for non-academic reasons had significantly lower PREview scores than did the baseline group, with a large effect size. uGPA was also significantly lower in the non-academically dismissed group and had a medium effect size. MCAT scores were comparable between these groups.

Conclusions

Medical student terminations have costly repercussions for a variety of constituents. To help mitigate termination outcomes, we sought to understand if common admissions metrics may be associated and thus potentially serve as useful predictors. Based on the preliminary evidence presented in this study, we conclude that metrics of academic readiness, such as MCAT scores and uGPA, are strong predictors of academic withdrawal and academic dismissal outcomes, whereas PREview scores, a metric of professional readiness, are a strong predictor of non-academic dismissal.

## Introduction

Termination from medical school is relatively rare and generally occurs through either withdrawal or dismissal. Reasons for termination are often multifactorial and complex, as students must deal with unanticipated life events, health issues, changing career interests, poor academic performance, and professionalism concerns, among other issues. The effects of a termination outcome are both consequential and far-reaching.

For students, the combination of substantial tuition costs and steep financial debt is often followed by pressure to begin immediate repayment of loans. Furthermore, terminated students typically have limited earning potential, which makes the debt-to-income ratio even more difficult to overcome. Such financial situations are likely to create considerable stress and anxiety, feelings of failure or shame, and potentially a decline in mental health [1]. For medical schools, student termination often results in an array of costs. Beyond financial losses, schools also experience a loss of return on human capital investments. Also, faculty and staff who provided emotional and resource support are often left frustrated and disappointed, sometimes contributing to turnover [2-3]. Additionally, schools with lower graduation rates may suffer reputational damage by receiving lower rankings in various national metrics (e.g., U.S. News & World Report) and find themselves under increased scrutiny from medical school accreditors (e.g., Liaison Committee on Medical Education (LCME)).

For the public, student termination reduces the future physician workforce, creating ripple effects throughout healthcare systems and communities. Fewer available physicians can lead to increased strain on the existing workforce, leading to longer waiting times to see a doctor, less time spent during a patient/physician encounter, and decreased quality of care [4]. Patients with reduced access to primary care may seek costly emergency department care, which further strains healthcare resources. [5-6]. For the existing physician workforce, student termination conveys a message that less help is on the way. Fewer physicians entering the workforce can lead to heavier workloads, a higher risk of burnout, and an increase in medical errors [6]. Consequently, overworked physicians may withdraw from the workforce by retiring early, seeking non-clinical employment, or changing professions [7].

Although the number of individuals terminated for academic and non-academic reasons annually is quite small, the collective impact of these losses is both extensive and severe. Thus, reducing student attrition wherever possible aligns with the best interests of all stakeholders. While some types of termination may be impossible to predict, those associated with academic performance or non-academic factors such as professionalism may be more foreseeable. However, little research has explored potential predictors of academic and non-academic termination in medical education. Accordingly, this study aimed to compare admissions metrics between students in MD degree-granting programs terminated for academic or non-academic reasons versus all other matriculating students and recent graduates.

## Materials and methods

Measures

The Medical College Admission Test (MCAT®) exam is a standardized test that measures an applicant’s academic readiness for medical school. The exam consists of four sections (Biological and Biochemical Foundations of Living Systems; Chemical and Physical Foundations of Biological Systems; Psychological, Social, and Biological Foundations of Behavior; and Critical Analysis and Reasoning Skills) with a total score ranging from 472 to 528. The Association of American Medical Colleges (AAMC) PREview® Professional Readiness Exam (henceforth, PREview) is a situational judgment test (SJT) that measures an applicant’s professional readiness for medical school. The exam is scored on a 9-point scale, with higher scores indicating better judgment in response to real-world professional challenges they may encounter in medical school with respect to relational skills (e.g., work effectively on teams, build relationships, compassionately engage with patients and colleagues, etc.) and personal accountability (e.g., resilience, ethical responsibility, adaptability, etc). Undergraduate grade point average (uGPA) is a standardized measure of grade point average across undergraduate institutions. Because applicants come from a variety of undergraduate institutions that use different grading systems and academic calendars, the American Medical College Application Service (AMCAS®) application service produces a standardized GPA measure ranging from 0 to 4 that allows medical schools to make objective comparisons of uGPA [8].

Procedures

This study examined aggregate national data for student cohorts matriculating into traditional MD-granting degree programs during the 2021-2024 academic years. We examined only those students who 1) withdrew for academic reasons; 2) were dismissed for academic reasons; or 3) were dismissed for non-academic reasons. Termination outcome data were collected as of September 2025. Thus, students matriculating in 2021 would have four years of data to examine, whereas students matriculating in 2022 would only have three years of data, students matriculating in 2023 would have only two years of data, and students matriculating in 2024 would have only one year of data.

A baseline group was established, which included all matriculants and graduates during this period but excluded anyone who took a leave of absence, withdrew, or was terminated for any other reason. Students must have both an MCAT score and a uGPA to be included in this baseline group. This resulted in a total of 78,176 baseline matriculants and recent graduates. Only the most recent MCAT scores, uGPA, and PREview scores were included in the analysis. Because the number of students terminated for these reasons was quite small (n = 404) and only a subset had PREview scores (n = 28,381), we first divided the students into two groups: those with PREview scores and those without PREview scores. We then compared MCAT scores and uGPAs between the groups to assess any significant differences. As no significant differences emerged (Table 1), the groups were merged into a single aggregate sample and compared with the baseline group on each measure.

**Table 1:** Comparison of academic metrics by presence or absence or PREview scores PREview: Professional Readiness Exam; MCAT: Medical College Admission Test; uGPA: undergraduate grade point average

Termination type	Group with PREview scores			Group without PREview scores			Difference	
	N	MCAT median	uGPA median	N	MCAT median	uGPA median	MCAT p-value	uGPA p-value
Withdrew - academic	28	508	3.66	141	507	3.70	0.81	0.65
Dismissed - academic	31	508	3.54	162	504	3.62	0.08	0.18
Dismissed - non-academic	13	511	3.57	29	511	3.77	0.70	0.10

Data analysis

Data analyses consisted of calculating descriptive statistics for MCAT scores, uGPA, and PREview scores for each termination outcome. Consistent with previous national studies examining MCAT scores and uGPA data, and because small sample sizes are more sensitive to extreme scores, we examined the median statistic for each metric [9-10]. We used a Mood’s median test with alpha set to 0.05, and Cliff’s delta effect sizes to determine the practical significance of any differences. Effect sizes were interpreted with values approximating 0.15 indicating a small effect, 0.33 a medium effect, and 0.47 a large effect [11]. All data were analyzed using Stata 18.0 (StataCorp, College Station, TX) statistical software.

Research ethics

All students included in this study provided consent for their data to be used for research purposes when they completed the AMCAS application, the MCAT® exam, and the PREview exam, all owned by the AAMC. This study was deemed exempt by the American Institutes for Research institutional review board (protocol: #2218897-1).

## Results

Results indicate that students who withdrew for academic reasons had significantly lower median MCAT scores and uGPAs compared to students in the baseline group. More specifically, students who withdrew for academic reasons had MCAT scores that were 5 points lower than the baseline, indicating a large practical difference (δ = 0.42). Median uGPAs were 0.14 points lower than the baseline, indicating a medium practical difference (δ = 0.34). Although PREview scores were not statistically significantly different from the baseline, the 2-point difference equated to a practical difference that was of medium size in magnitude (δ = 0.31) (Table 2).

**Table 2:** Comparison between termination outcomes groups and baseline group MCAT: Medical College Admission Test; uGPA: undergraduate grade point average; PREview: Professional Readiness Exam

Termination type	N	MCAT median	Cliff’s δ	uGPA median	Cliff’s δ	PREview median	Cliff’s δ
Withdrew - academic	169	507^*^	0.42	3.70^*^	0.34	4	0.31
Dismissed - academic	193	505^*^	0.48	3.59^*^	0.49	6	0.18
Dismissed - non-academic	42	511	0.21	3.69^*^	0.32	3^*^	0.51
Baseline (matriculants/graduates)	78,176	512		3.84		6	

Similarly, students who were dismissed for academic reasons also had significantly lower median MCAT scores and uGPAs compared to the baseline group. However, the contrast in median MCAT scores was more pronounced with a 7-point difference, indicating a large practical difference (δ = 0.48). Furthermore, median uGPAs were also more pronounced with a 0.25-point difference, indicating a large practical difference (δ = 0.49). Median PREview scores were identical to students in the baseline group.

Students dismissed for non-academic reasons had comparable median MCAT scores to the baseline group with no statistically significant differences. However, median uGPAs were significantly lower (0.15 points) than the baseline group, indicating a medium practical difference (δ = 0.32). The PREview scores were also significantly different, as the group dismissed for non-academic reasons had median scores three points lower than the baseline group. This contrast indicated a large practical difference (δ = 0.51).

## Discussion

Students who withdrew for academic reasons had significantly lower MCAT scores and uGPAs compared to students in the baseline group. The practical differences measured by effect sizes were large for MCAT scores and medium for uGPA. The PREview scores, although not statistically significantly different, did present a medium effect size, indicating a practical difference that was medium in magnitude. The current study did not specifically examine why MCAT scores had a greater association with academic withdrawal than uGPA. However, recent national studies have consistently shown MCAT scores are better predictors of academic performance in medical school than uGPA, leading us to speculate that the stronger association is due to MCAT scores being a more direct and standardized measure of premed academic readiness [9-10].

Regarding why uGPA and PREview scores, two very different measures, may also provide some predictive benefits, we speculate that uGPA may encompass elements of various non-cognitive factors such as work ethic, conscientiousness, and persistence, whereas PREview scores may be a good indicator of factors such as resilience, adaptability, and continuous self-improvement. Because median PREview scores were lower than the baseline group, we also speculate that those who withdrew likely experienced a combination of academic and professional issues. Additional research is warranted to gain more insights into this area.

Students dismissed for academic reasons also had significantly lower median MCAT scores and uGPAs than the baseline group, and the contrast in scores was large in magnitude for both measures. The PREview scores were identical between the terminated and baseline groups, indicating no meaningful difference. Thus, our findings suggest that MCAT scores and uGPA are predictive of academic dismissal outcomes, whereas PREview scores are not. We speculate that MCAT scores may be a good indicator of academic propensity specific to medical school, whereas uGPA may be a good indicator of academic persistence and general academic adaptability. As both academic metrics were significantly lower, it may be indicative of all-around academic underpreparedness. Because PREview scores indicated no difference, we speculate that students dismissed for academic reasons primarily experienced academic difficulties. Additional research is also warranted in this area.

The large and statistically significant contrast in median PREview scores between those dismissed for non-academic reasons and the baseline group indicates that PREview scores are predictive of non-academic problems. Given that PREview scores provide a measure of professional readiness, we find this link unsurprising, since dismissal for non-academic reasons is often associated with underlying professionalism issues and behaviors. MCAT scores, however, were comparable between the groups, indicating minimal, if any, predictive value for non-academic dismissal.

The uGPA between the two groups differed by 0.15 points, which indicated a significant effect of a medium magnitude. Thus, there is some evidence to suggest that uGPA may also have some predictive value for this outcome. We speculate that the reason uGPA may be a predictor of non-academic dismissal, whereas MCAT scores are not, may be due to the differences in signals each measure provides. That is, MCAT scores are specific to academic readiness for medical school, whereas uGPA provides a more global indicator of academic readiness that could potentially capture additional factors such as persistence, work ethic, etc.

Limitations

This study has several limitations. First, we investigated data beginning with matriculating students from the 2021 cohort and captured a snapshot of data as of September 2025. Only one cohort of students (2021) completed four years of medical school during this time. Moreover, because it has become increasingly common for students to take five years to complete a traditional four-year program, some students from the 2021 cohort and all students from the 2022-2024 cohorts are still eligible to withdraw or be dismissed for some reason in future datasets. Therefore, follow-up analyses that incorporate cohorts with fully completed four- to five-year timelines will be important for future research.

Second, although this study includes national data for the MD student population between 2021 and 2024, the number of students with termination outcomes is incredibly small relative to the number of students who are successful each year. Furthermore, there were many students within this small population without PREview scores, which further limited the sample size for this study. As described earlier, to mitigate the risks of potential sample bias, we compared metrics based on groups with and without PREview scores and found minimal differences between the groups despite the missing data on PREview. While we find this reassuring, future studies will require larger samples with more complete PREview data.

Finally, we are unsure of the extent to which classification errors may exist within the data. In this study, students’ last status codes as reported by medical school registrars were used to determine termination outcomes. It is unknown how many students, if any, may ultimately re-enter medical school at a later date. Additionally, medical school administrators often feel an ethical obligation to provide face-saving, compassionate off-ramps for students [12]. Thus, it is possible, for example, that some students who otherwise may have been dismissed for academic reasons were given an opportunity to withdraw instead. Nonetheless, we believe that dismissal cases (whether academic or non-academic) are likely to involve minimal classification error.

## Conclusions

Medical student terminations carry high costs for multiple stakeholders. To help mitigate termination outcomes, we sought to understand if common admissions metrics may be associated and thus potentially serve as useful predictors. Using a national dataset from the 2021 to 2024 academic years, we examined potential associations by comparing MCAT scores, uGPAs, and PREview scores for termination outcome groups against a baseline of non-terminated matriculants and recent graduates. Findings revealed several statistically significant differences with medium and large effect sizes. Based on this preliminary evidence, we conclude that metrics of academic readiness, such as MCAT scores and uGPA, are strong predictors of academic withdrawal and academic dismissal outcomes, whereas PREview scores, a metric of professional readiness, are a strong predictor of non-academic dismissal.
